# Economical analysis of saturation mutagenesis experiments

**DOI:** 10.1038/srep10654

**Published:** 2015-07-20

**Authors:** Carlos G. Acevedo-Rocha, Manfred T. Reetz, Yuval Nov

**Affiliations:** 1 Department of Organic Synthesis, Max-Planck-Institut für Kohlenforschung, Mulheim, 45470, Germany; 2 Department of Chemistry, Philipps-Universität Marburg, 35032, Germany; 3 Department of Statistics, University of Haifa, Haifa, 31905, Israel; 4 Prokaryotic Small RNA Biology Group, Max-Planck-Institut für terrestrische Mikrobiologie, Marburg, 35043, Germany; 5 Landes-Offensive zur Entwicklung Wissenschafltich-ökonomischer Exzellenz (LOEWE) Centre for Synthetic Microbiology (SYNMIKRO), Philipps-Universität Marburg, 35032, Germany

## Abstract

Saturation mutagenesis is a powerful technique for engineering proteins, metabolic pathways and genomes. In spite of its numerous applications, creating high-quality saturation mutagenesis libraries remains a challenge, as various experimental parameters influence in a complex manner the resulting diversity. We explore from the economical perspective various aspects of saturation mutagenesis library preparation: We introduce a cheaper and faster control for assessing library quality based on liquid media; analyze the role of primer purity and supplier in libraries with and without redundancy; compare library quality, yield, randomization efficiency, and annealing bias using traditional and emergent randomization schemes based on mixtures of mutagenic primers; and establish a methodology for choosing the most cost-effective randomization scheme given the screening costs and other experimental parameters. We show that by carefully considering these parameters, laboratory expenses can be significantly reduced.

Following the seminal work of Michael Smith concerning site-directed mutagenesis^1^, saturation mutagenesis (SM) has emerged as an indispensable technique in molecular biology for introducing targeted sequence variations at virtually any DNA region. Areas of application include laboratory evolution of enzymes with enhanced activity, stability, and stereoselectivity^2^,^3^,^4^,^5^,^6^, manipulation of binding properties of antibodies^7^ and transcription factors^8^. More recently, SM has been used to engineer promoters^9^, transcriptional enhancers^10^, ribosome binding sites^11^, *cis*-regulatory elements and *trans*-acting factors^12^, and protein-coding genes using emergent genome engineering tools^13^. Consequently, SM, especially when applied iteratively^3^,^4^,^5^,^14^,^15^, has become an effective tool in protein engineering^2^,^4^,^5^, and holds great potential in the directed evolution of metabolic pathways^16^,^17^,^18^ and genomes^19^,^20^,^21^.

However, creating high-quality SM libraries is still an experimental challenge^6^: Factors such as the target DNA sequence, G+C content, melting-temperature of DNA duplex, randomization scheme, primer quality, length, and annealing, all play a role in the quality of the resulting library^22^,^23^,^24^. While some of these factors are inherent to the DNA target sequence, and thus cannot be easily modified, other factors, such as the randomization scheme and primer quality, are decided upon by the experimenter. Yet these decisions carry their costs: more or higher-quality primers, for example, are obviously more expensive. To our knowledge, no study has linvestigated systematically the decision-making involved in SM experiments from the economical perspective.

In many directed evolution settings, genetic selection of the randomized variants is not an option, so that expensive screening must be undertaken instead^25^. In these cases, the screening effort increases exponentially as a function of the number of randomized positions. To alleviate this burden, it is advantageous to minimize both redundancy and the frequency of premature stop codons. One step in this direction is to use NNK or NNS degenerate codons (where N = A/C/G/T, K = G/T and S = C/G) instead of NNN (but see^26^); this way, when randomizing four positions, the theoretical ratio between the most represented variants (with Arg/Leu/Ser at each randomized position) and the least represented ones (with Met/Trp) drops from 1296:1 to 81:1, and the frequency of prematurely truncated variants drops from 17.5% to 11.9%. However, it is not possible to reduce further the redundancy using a single degenerate primer, while still encoding all 20 amino acids^27^, so other strategies are called for.

Four such strategies have emerged. The first uses specially prepared mono^28^, di^29^, or trinucleotides^30^ phosphoramidite solutions, or combinations thereof^31^, for synthesizing redundancy-free mutagenic primers. A second strategy, dubbed MAX, relies on the synthesis of a “template” and twenty “selection” oligonucleotides and their hybridization, and shows that library redundancy can be completely eliminated^32^; this method has been recently extended to contiguous codons by using ProxiMAX^33^. A third strategy builds on solid-phase combinatorial gene synthesis, and results in libraries with the highest diversity and essentially no bias^22^. Yet in spite of their clear advantages, these three strategies are currently too expensive to be used in routine SM experiments.

The fourth strategy, which we explore in this work, uses mixtures of ordinary primers to achieve zero or near-zero redundancy: Tang *et al*.^34^ showed that a mixture of four primers per randomized position, NDT, VMA, ATG, and TGG (where D = A/G/T, V = A/C/G, M = A/C) at 12:6:1:1 molar ratio, results in a zero probability for premature stop codons and a perfectly uniform theoretical distribution of 1/20 for each of the 20 amino acids; this scheme is referred to hereafter as “Tang”. The “22c-trick” approach by Kille *et al*.^35^ uses even fewer primers per randomized position – namely, the three primers NDT, VHG, and TGG (where H = A/C/T), at 12:9:1 molar ratio – and results also in a zero probability for premature stop codons, and in an almost uniform amino-acid distribution: 2/22 for Leu and Val, and 1/22 for each of the remaining 18 amino acids. More sophisticated variations of the primer mixing strategy have been published recently^36^,^37^,^38^.

The last strategy (Tang and 22c-trick) is the most accessible to any molecular biology lab. However, this strategy requires more primers than NNK and NNS, which means that it is worthwhile to use it only when the screening cost is high enough, so that the reduction in screening effort due to the smaller library size more than offsets the increased library generation cost due to the larger number of primers. An even more extreme option is to discard randomization altogether, and to produce individually, via site-directed mutagenesis, all possible variants; the screening cost here would be minimal, as no duplicates are screened, but mutagenesis will be most expensive, because each variant requires a separate PCR reaction with dedicated primers.

Regardless of the mutagenesis approach chosen, it is expedient to assess experimentally, before engaging in costly library screening, whether the desired DNA diversity has indeed been created, to avoid wasting resources. For this reason we introduced some time ago the semi-quantitative Quick Quality Control (QQC) – a single, pooled sequencing reaction of the library’s plasmids, which provides an early indication for whether the randomization was successful^24^,^35^,^39^,^40^. Recently, Stewart and colleagues have extended this simple and fast control by introducing the Q-value, an attractive and user-friendly quantitative score that averages the QQC results for estimating overall randomization efficiency^23^.

The present study is concerned with all the above *economical* aspects associated with SM. Firstly, we present a cheaper and faster manner to perform the QQC. Secondly, we study how primer purity influences library quality. Thirdly, we assess and compare library quality using primers from three different suppliers. Fourthly, we compare library costs and quality using various redundant and non-redundant randomization schemes. Fifthly, we analyze the bias in favor of the wild-type (WT) codon and its implication on the expected library diversity. Finally, we present a methodology for resolving the trade-off between the cost of library generation and the cost of library screening, i.e., for choosing optimally the randomization scheme given the various experimental parameters.

## Materials and Methods

### Saturation mutagenesis experiments

The gene coding for P450_BM3_ was cloned into the pRSF-Duet-1 vector (Novagen, Merck, Darmstadt, Germany) under the control of the T7 promoter as described earlier^41^. The forward 5′-CGCTTTGATAAAAACTTAXXXCAAGCGCTTAAATTTGTACGTG-3′ and reverse 5′- CACGTACAAATTTAAGCGCTTGZZZTAAGTTTTTATCAAAGCG-3′ primers (where XXX and ZZZ respectively denote NNK or NNS or NDT+VHG+TGG or NDT+VMA+TGG+ATG and MNN or SNN or AHN+CDB+CCA or AHN+TKB+CCA+CAT) in both desalted (gel filtration column) and HPLC-purification grade, were purchased in Metabion (Martinsried, Germany; supplier 3) and delivered in water at a concentration of 100 μM. The same primers for NNK randomization in the same two purity grades and final concentration were acquired from Integrated DNA technologies (Iowa, US, supplier 1) and Invitrogen or Life Technologies (Carlsbad, US; supplier 2). All primers and primer mixtures were diluted to a concentration of 10 μM. The primer ratios for the 22c-Trick and Tang libraries were mixed as follows: 12 eq. NDT + 9 eq. VHG + 1 eq. TGG, and 12 eq. NDT + 6 eq. VHA + 1 eq. TGG + 1 eq. ATG, respectively. Prior to PCR, 1 μL (10 pmoles) of each forward and reverse primer or primer mixture were mixed with 20 ng of plasmid template in 8 μL of water, plus 10 μL of 1:1 KOD Hot Start Master Mix (Novagen, Merck, Darmstadt, Germany). The following PCR conditions were set: 95 °C for 2 min, followed by 25 cycles at 95 °C for 20 sec, 50 °C for 30 sec and 70 °C for 4 min, ending with 70 °C for 5 min and subsequent cooling. The samples were then dialysed against Millipore-Q water using 0.05 μm Millipore MF membrane filters (Millipore, Merck, Darmstadt, Germany) for 30 min. The methylated template was then treated with 1 μL (20 units) *DpnI* (New England Biolabs, Ipswich, US) for at least 48 h at 37 °C in appropriate buffer and dialysed as before for 30 min prior to electroporation. From these dialysed samples, 5 μL were used to transform 50 μL of *Escherichia coli* BL21-Gold(DE3) cells using a “MicroPulser” electroporator (BioRad, Hercules, US). The cells were resuspended in 945 μL of LB medium without antibiotic and recovered for 1 h at 37 °C and 220 rpm in a 14 mL Falcon tube. Finally, the 1000 uL of culture were either plated on a big (15 cm diameter) agar plate with kanamycin (50 μg/mL) or left in the tube, to which 4000 μL of LB medium with 60 μg/mL kanamycin were added, resulting in 5 mL LB broth with 48 μg/mL kanamycin. The cultures were incubated at 37 °C with (liquid cultures) or without (solid agar plates) shaking at 220 rpm overnight (for about 16 h).

### QQC, Q-values, and sequencing

The Quick Quality Control (QQC) based on solid agar plates was performed as previously described^22^. Briefly, upon growth, colonies were harvested using a Drigalsky spatula upon addition of 2 mL of pure water. The plasmid DNA was then extracted from the collected cells using the QIAprep Miniprep Kit (Qiagen, Hildesheim, Germany) and sequenced using a T7-coding primer. For QQC based on liquid cultures, the cells from the 14 mL Falcon culture tube were centrifuged for 10 min at 4000 rpm, followed by extraction of the pooled plasmids and sequencing.

To estimate the base distribution at each base position of the randomized codon, the peak heights of the four chromophores representing adenine (A), thymine (T), guanidine (G) and cytosine (C) were obtained by moving the mouse cursor over the peaks using the freely available program “Chromas Lite” (Technelysium Pty Ltd, South Brisbane, Australia). The values were then converted to pie diagrams using Microsoft Excel (V. 14.3.2, Microsoft Corporation) and compared to the expected values corresponding to the randomization scheme: NNK or NNS expect 25% of A/T/G/C (N) at the first and second base positions, and 50% of G/T (K) or C/G (S) at the third position. 22c-Trick expects 27%(A)+19%(T)+27%(G)+27%(C) at the first position, 32%(A)+32%(T)+18%(G)+18%(C) at the second, and 55%(T)+45%(G) at the third^35^. Finally, Tang expects 30%(A)+20%(T)+25%(G)+25%(C) at the first position, 35%(A)+25%(T)+20%(G)+20%(C) at the second, and 30%(A)+60%(T)+10%(G) at the third^34^. The pooled Q-values, Q_pool_, were calculated from the base distribution percentages, and the experimental Q-values, Q_exptl_, were calculated from the sequencing results, as described elsewhere^23^.

Upon analyzing library quality, the plasmid sent for QQC can be used to transform newly competent cells. In this case, the same strain *E. coli* BL21-DE3(Gold) was transformed as indicated above, using 50 ng of the plasmid isolated from the agar plate, but only 50 μL of the 1000 μL cell suspension were plated on big LB agar plates, followed the next day by single colony picking into 400 μL of LB medium with appropriate antibiotic (kanamycin at 50 μg/mL), using 2.2 mL 96-well plates (Thermo Scientific Fischer, Waltham, US). The plates were incubated at 37 °C and rotated at 220 rpm overnight; the next day an aliquot of 10 μL was transferred to 96-well solid agar plates with proper antibiotics and sent for plasmid extraction and sequencing using the T7-coding primer (GATC, Konstanz, Germany).

### Statistical analysis

All statistical analyses were carried out using the R software (http://www.r-project.org). Correlation was estimated through Pearson’s product moment correlation coefficient, and Q-values were compared using Wilcoxon signed-rank test. Yields were compared using a two-sample proportion test. Point estimates and confidence intervals for P_WT_ were computed via a binomial test. The goodness-of-fit tests for annealing uniformity are based on Pearson’s chi-squared test statistic *x*^2^ = Σ_*i*_(O_*i*_*−E*_*i*_)^2^/E_*i*_. Because of the low number of observations (sequencing results) relative to the number of categories (possible codons), the distribution of the χ^2^ statistic cannot be approximated by the asymptotic chi-squared distribution, and hence the p-values were computed using a Monte Carlo test with 5,000 replicates. Results were considered significant when p < 0.05. No adjustments were made for multiple testing.

The library sizes required under the various scenarios – L_NNK_, L_NNS_, L_22c_, and L_Tang_ – were computed by TopLib (http://stat.haifa.ac.il/~yuval/toplib)^42^.

## Results

The model protein used in this study is the mutant F87G of the cytochrome P450_BM3_ from *Bacillus megaterium*, a self-sufficient and highly active monooxygenase that displays an extraordinary catalytic diversity on many natural and non-natural substrates^43^. In further mutagenesis work, we chose to target residue Ser72 (S72, WT codon: AGT) because it has been recognized to play an important role in the selectivity of P450_BM3_ towards steroids^44^, a challenging synthetic reaction in organic chemistry^40^.

In total, we generated 12 SM libraries in 12 separate experiments. The experiments differed in the randomization scheme used (NNK / NNS / 22c-trick / Tang), primer supplier and primer purity (desalted/HPLC). See Table 1. The QuikChange protocol^45^,^46^ was used in all cases but employing a different polymerase from the traditional protocols (see Material and Methods).

After transformation and recovery, cells from each of the 12 libraries were grown in solid and liquid media containing the proper antibiotic. The next day, cells from each of the 24 resulting cultures were harvested and lysed, followed by extraction and sequencing of the pooled plasmids to perform the QQC and determine the Q_pool_ values. Next, a single 96-well plate was sequenced for each library using the samples obtained from the solid agar plates; based on the sequence data, the experimental QQC-like charts and Q_exptl_ values were determined.

### Quick quality control (QQC) and Q-values

The purpose of QQC is to assess whether the desired diversity was introduced at the target codon, by observing the percentage of bases at each of the three base positions of the codon using a single, pooled DNA sequencing electropherogram. These percentages were converted (see Methods) to a Q-value, a number ranging from Q = 0, indicating no randomization, to Q = 1, indicating perfect randomization^23^. The QQC charts and the Q-values are reported in Table 1.

The Q_pool_ values obtained under the two culturing conditions (solid vs. liquid) were highly correlated (r = 0.74, p = 0.006), with no statistically significant difference in magnitude between conditions (p = 0.20). This finding indicates that QQC under the two conditions carries similar information, so that the two procedures are exchangeable. No statistically significant correlation was found between the Q_pool_ values and the Q_exptl_ values, neither for solid nor for liquid media. There was also no statistically significant difference in the Q_pool_ values when comparing libraries according to purity grade (desalted vs. HPLC).

When comparing the six NNK libraries, the ones based on primers from supplier 3 exhibited the highest mean Q_pool_ value, compared to suppliers 1 and 2 (0.738 vs. 0.603 and 0.507, respectively). Therefore, we used only primers from supplier 3 to compare the three other randomization schemes: NNS, Tang and 22c-trick.

### Analysis of sequencing data

A small number of the samples (≤4 per library) yielded low-quality sequencing results, a common finding observed in other studies^22^,^23^. Of the successfully sequenced variants, some exhibited imperfect randomization due to various reasons: Additional mutations outside of the target position, misplaced insertion of the primer within the coding sequence, presence of two or more bases at one or more positions of the target codon, etc. See Table 2. We use the term “yield” to denote the percentage of the successfully randomized variants (including those carrying the WT codon) out of the successfully sequenced ones.

The purity of the primers plays a crucial role in determining library yield: The desalted primers exhibited a significantly lower yield than the HPLC ones (mean yield 56.6% vs. 66.7%, respectively; p < 0.001), except for supplier 1 with a yield of 72.3%. In fact, within the libraries based on desalted primers, the yield of the library corresponding to supplier 1 was higher than that of the two other suppliers (p = 0.0013); no statistically significant differences in yield between suppliers were found within the libraries based on HPLC primers.

Three of the twelve libraries included all 20 amino acids, whereas the remaining nine libraries missed up to 4 amino acids. The Q-value of the liquid media libraries was negatively correlated with the number of amino acids missed (r = −0.58, p = 0.046), indicating that the Q-value in such libraries possesses a predictive power regarding final amino-acid diversity. No similar statistically significant correlation was found in the solid media libraries, and no correlation was found between the Q-values and yield (in either solid or liquid media).

**Figure 1:**
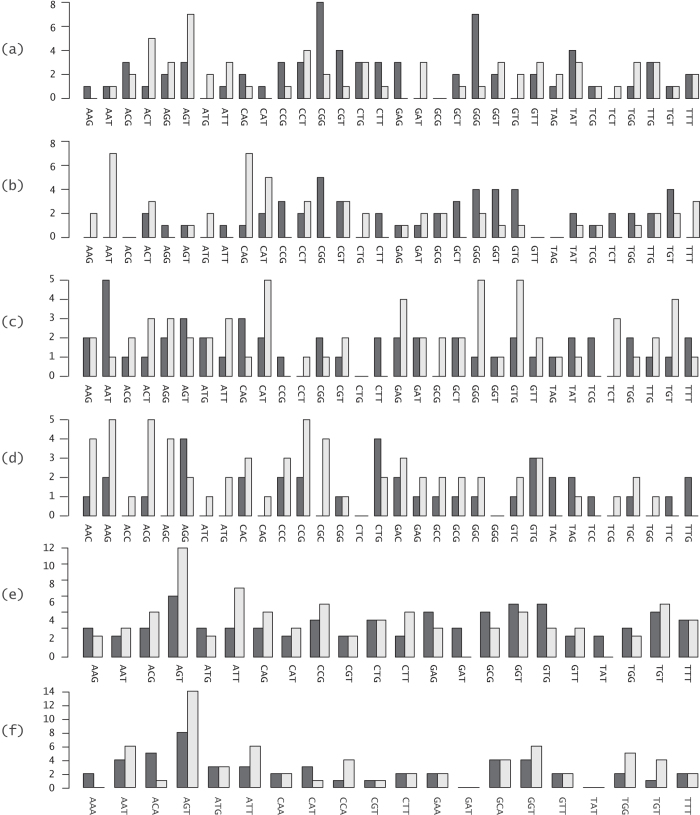
Sequencing results of randomized position S72 (WT codon: AGT) obtained from single colonies formed on agar plates. Vertical axes denote counts (how many codons of each type were found in the sequencing). Black columns denote desalted primers, and grey ones denote HPLC primers. Libraries: (**a**) 1-2; (**b**) 3-4; (**c**) 5-6; (**d**) 7-8; (**e**) 9-10; and (**f**) 11-12.

The prevalent assumption used in mathematical analyses of SM experiments is that the randomized codon distribution is uniform, i.e., each of the possible codons (32 codons in NNK and in NNS, 22 in 22c-trick, and 20 in Tang) is equally likely to anneal to the template and form a variant^42^,^47^,^48^,^49^. An alternative assumption, which we now examine, is that the distribution is not uniform, and in particular, that there is a bias in favor of the WT codon. Figure 1 shows the distribution of the randomized codons for each of the 12 libraries. Statistical analysis reveals a statistically significant bias in favor of the WT codon, AGT, in five of the libraries: HPLC NNK from supplier 1 (p = 0.004), the two 22c-trick libraries (p = 0.044 for desalted and p < 0.0001 for HPLC), and the two Tang libraries (p = 0.004 for desalted and p < 0.0001 for HPLC). In all of these cases, the randomization resulted in AGT with likelihood significantly higher than expected under a uniform distribution. Since AGT is not among the 32 NNS codons (and indeed, no AGT codon was sequenced in any of the two NNS experiments), the two NNS experiments were excluded from this analysis. In the five remaining libraries, no statistically significant evidence for deviation from uniformity was detected, but the confidence intervals for the probability of WT annealing, P_WT_, across libraries are too wide and overlapping to rule out the possibility of WT bias also in these cases.

In all but two of the twelve libraries, the annealing distribution among the remaining, non-WT codons does appear to be uniform. The exceptions that exhibited statistically significant deviation from uniformity were library 1 (p = 0.016) and library 4 (p = 0.002).

### Cost effectiveness of randomization schemes

Throughout the following analysis, we assume that the QuikChange protocol is used, or a variation thereof employing two partially-overlapping mutagenic primers that may be more suitable for some templates^50^, and that the designer’s goal is 95% expected coverage of protein space. This metric, which is sometimes called “95% fractional completeness”, is equivalent to requiring a 0.95 probability for discovering the best variant in a given protein sequence space^42^. The results remain qualitatively unchanged under other library metrics, e.g., requiring 90% expected coverage, or replacing expected coverage with the probability of discovering at least one of the top three variants^42^.

We consider first an idealized SM experiment of a single protein position, in which the yield is 100% and there is no annealing bias. When using NNK (or NNS) randomization, the total cost of the experiment is:


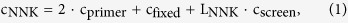


where c_primer_ is the per-reaction cost of a *single* primer, c_fixed_ is the sum of all other fixed costs associated with a single PCR reaction (labor, dNTPs, polymerase, buffer, template DNA, DpnI, transformation), c_screen_ is the screening cost of a single variant, and L_NNK_ = 80 is the number of variants screened (the library size) required to achieve 95% expected coverage^42^. Similarly, under NNN, 22c-trick, and Tang randomization, the total experimental costs are:


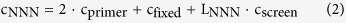



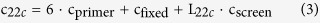



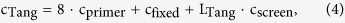


where L_NNN_ = 92, L_22c_ = 63, and L_Tang_ = 59. When generating each of the 20 variants individually via site-directed mutagenesis, 20 PCR reactions are required with a pair of primers in each, so the total cost is:


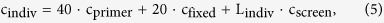


where L_indiv_ = 20.

To eliminate the dependence on the currency used, we set c_fixed_ = 1 and measure all costs as multiples of c_fixed_. This allows focusing on the relative magnitude of the prices, rather than on their actual values, which change rapidly as a result of technological advances.

**Figure 2:**
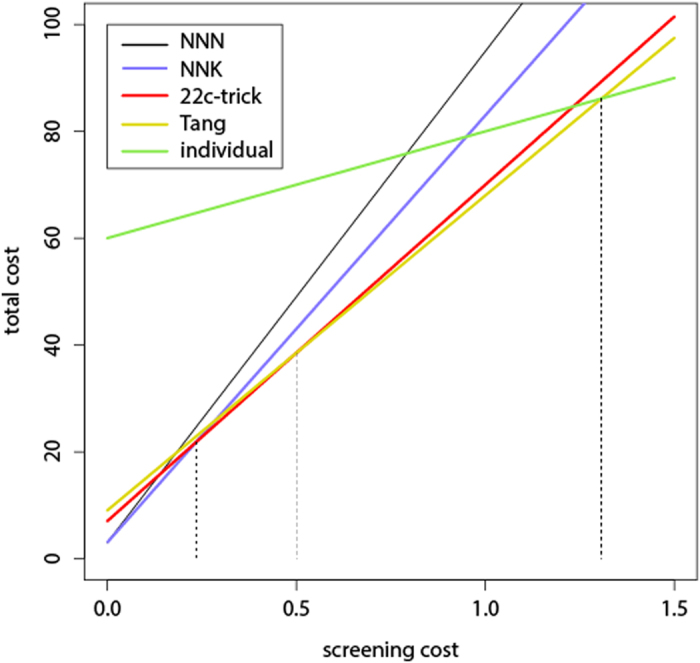
Total cost as a function of screening cost, when randomizing a single position using 5 randomization schemes. Primer cost is c_primer_ = 1.

Figure 2 shows the total cost as a function of c_screen_, the screening cost, while assuming c_primer_ = 1. The five straight lines correspond to the five randomization schemes. For each value of the screening cost (an x value), we are interested in the total cost (y value) that is minimal among the five schemes. These minimal values constitute the so-called *lower envelope* of the five lines. It can be seen that when 0 ≤ c_screen_ ≤ 0.24 the cost is minimized with NNK, when 0.24 ≤ c_screen_ ≤ 0.5 the cost is minimized with 22c-trick, when 0.5 ≤ c_screen_ ≤ 1.31 the cost is minimized with Tang, and when c_screen_ ≥ 1.31 it is most economical to generate the 20 variants individually. Note that NNN is never the randomization method of choice.

The differences in cost between 22c-trick and Tang are not very large. On the other hand, the differences between NNK and either 22c-trick or Tang are more substantial, and may exceed 30%. Generating the 20 variants individually becomes the optimal scheme only when screening cost is very high, but when this happens, this method may be considerably cheaper than all other schemes by a large margin.

**Figure 3:**
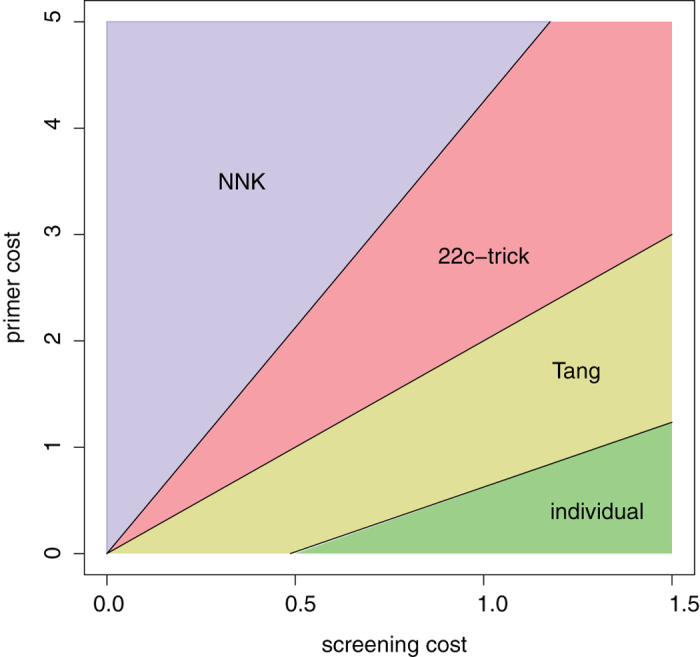
Cost space partitioned into regions according to the optimal randomization scheme (a single randomized position, assuming 100% yield, and no WT bias).

In Fig. 2 it was assumed that the primer cost equals the fixed cost. Of greater interest is to see how changes in both primer cost and screening cost (relative to the fixed cost of the reaction) determine the optimal randomization scheme. Figure 3 depicts how cost space – which is the two-dimensional plane, whose axes are the primer cost and screening cost – is partitioned into mutually exclusive regions, each corresponding to a different optimal randomization scheme. NNK is optimal when the screening cost is lower than the primer cost by a factor of 4.25 or more (this is the slope of the line separating the NNK and 22c-trick areas); as the screening cost increases, 22c-trick becomes optimal, then Tang, and finally individual generation of the 20 variants.

**Figure 4:**
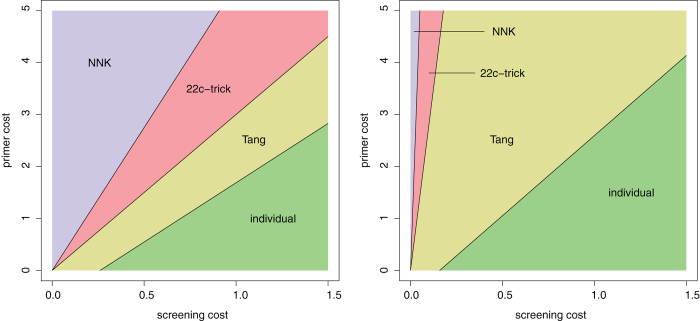
Cost space partition under 68% yield and WT bias P_WT_ = 0.12. Left: a single randomized position; right: two randomized positions.

The partition depicted in Fig. 3 changes under the more realistic assumptions of imperfect yield and some WT bias. The left panel of Fig. 4 shows the partition when the yield is changed from the ideal 100% to 68% (the average yield across the HPLC scenarios), and under a WT bias P_WT_ = 0.12 (the average bias across the scenarios). These changes result in an increased required library sizes: L_NNK_ = 121, L_22c_ = 99, and L_Tang_ = 93. The relative position of the four regions corresponding to the four schemes remains the same as in Fig. 3, but the exact location of their boundaries changes. The final total cost under the optimal scheme changes also: for example, when c_primer_ = 2.5 and c_screen_ = 0.75 (the middle point of the region depicted in Figs. 3 and 4 (a), for which 22c-trick happens to be the optimal scheme in both cases), the total cost is 76.0 when assuming 100% yield and no WT bias (as in Fig. 3), and 90.25 when assuming 68% yield and P_WT_ = 0.12 (Fig. 4 (a)).

Under uniform annealing distribution, NNK and NNS randomization are completely identical in terms of the resulting coverage of protein space, as they induce the same distribution over the 20 amino acids and the stop codon. However, when assuming WT bias, differences may occur, which depend on the specific randomized codon. The WT codon in our case is AGT, which is one of the NNK codons but not one of the NNS ones. Thus, under NNS randomization, the WT bias is avoided in this case, and the required library size (assuming 68% yield) is L_NNS_ = 118, slightly lower than the above reported L_NNK_ = 121. Even though NNS randomization is slightly more efficient than NNK in this case, we neglect this difference, and do not include NNS randomization in the analysis.

Another factor that influences the partition of cost space is the number of positions randomized (which was hitherto assumed to be 1). The right panel of Fig. 4 shows the partition of cost space when randomizing two positions, again under 68% yield and P_WT_ = 0.12. It is assumed that the two randomized positions are close enough on the primary sequence so that a single primer covers them. The number of NNK primers remains therefore 2, the number of 22c-trick primers is 2·3^2^ = 18, the number of Tang primers is 2·4^2^ = 32, and the number of primers required when generating individually each of the 20^2^ = 400 variants is 2·20^2^ = 800. The required library sizes also increase significantly, to L_NNK_ = 4881, L_22c_ = 3309, and L_Tang_ = 2917.

When compared to Fig. 3, a prominent feature of both panels of Fig. 4 is the smaller area of the NNK region. It can be shown mathematically that this is a general phenomenon, and that this region gets smaller as either the yield decreases or the number of randomized position increases.

## Discussion

The common thread in this work is the view of saturation mutagenesis experiments from the economical perspective.

The simple and cheap QQC was developed as a cautionary step to avoid wastage of resources in futile library screening^24^,^35^,^39^,^40^. When the QQC is performed in solid media, some colonies grow faster than others, creating a “colony bias”. To mitigate this problem, we recommend using liquid cultures, which also represent an economical advantage, since they require as little as a few mL (in our case 4 mL were added to 1 mL of cells in recovery medium) instead of 40 mL in the big solid agar plate format. This way, media costs could be decreased 10-fold, provided that a 14 mL Falcon tube for liquid cultures costs about the same as a large Petri dish. In addition, processing liquid cultures requires less time (only one centrifugation step) than processing solid-agar plates (cell harvesting plus centrifugation step), and the latter approach also requires more incubation space and workload. Thus, the liquid media QQC could be more useful for SM experiments in a high-throughput setting. Stewart and colleagues introduced the Q-value, which summarizes in a single number the QQC results^23^. Our statistical analysis shows that the Q-values obtained under the two culture conditions are highly correlated, but that only the liquid Q-value was correlated with the final amino-acid diversity. This finding suggests that the liquid QQC mitigates the “colony bias” problem present in the solid QQC format.

Another economical factor in SM experiments is primer cost. HPLC-purified primers generally cost up to 3 times more than column-purified desalted ones. To our knowledge, only two studies have indicated the role of primer purity in saturation or site-directed mutagenesis. Steffens and Williams reported that desalted primers were successfully used to create 200 single-site SM libraries of a polymerase, but no sequencing data of the resulting library mutants was provided^51^. On the other hand, the employment of HPLC-purified primers is recommended for the traditional QuikChange protocol, first by Stratagene^45^ and currently by Agilent^46^, the most widely used method for site-directed mutagenesis and SM^52^. Although no statistically significant difference in Q-value was found between desalted and HPLC libraries, our sequencing data shows that primer purity plays a critical role, with HPLC libraries enjoying significantly higher yield on average than desalted ones. Nevertheless, using desalted primers obtained from supplier 1 gave comparable results in terms of yield to HPLC-purified primers regardless of supplier, indicating that supplier 1 has higher primer quality standards than the two other suppliers regarding non-purified primers.

To further improve the economical efficiency of SM experiments, it is worthwhile to consider alternatives to the traditional NNK and NNS randomization schemes. We^35^ and others^34^ recently showed that the respective 22c-trick and Tang strategies can reduce the screening effort of single-site libraries compared to NNK/S, by a factor of about 50%. Other researchers have also demonstrated the practical utility of the 22c-Trick^53^,^54^ and Tang^55^,^56^ approaches. However, these “smarter” schemes entail a higher primer budget, and for this reason we established a methodological framework for comparing the cost-effectiveness of the various alternatives. The cost equations we use are perhaps somewhat simplistic, yet they capture the main trade-off between the cost of primers and the cost of screening: by switching to another randomization scheme, one can reduce the former by increasing the latter, or vice versa. The above analysis becomes important especially in large-scale, expensive experiments. This happens either when a large number of a protein’s positions are subject separately to SM, or when relatively many (say, ≥3) positions are randomized simultaneously. In either case, we recommend that a short, preliminary study be conducted before the main one, to provide estimates for the model parameters (yield, WT bias) required for choosing optimally the randomization scheme and the library sizes under the specific experimental conditions at hand.

We show that under certain experimental settings – namely, when screening cost is high enough – the 22c-trick and Tang schemes are economically superior alternatives to NNK and NNS. Perhaps surprisingly, this is true also when several close-by positions are randomized simultaneously. Indeed, the number of primers required for 22c-trick or Tang randomization grows exponentially as the number of randomized positions increases, but the saving in screening efforts due to the reduction in library size (relative to NNS or NNK) more than offsets the increased cost of primers. When choosing between NNK and NNS, we recommend choosing the scheme that does not include the template’s WT codon (as was NNS relative to the WT codon AGT in our case), in order to avoid the WT bias problem and to get a more uniform annealing distribution. The difference in the resulting library size, and hence in screening cost, may be small or even negligible (as was in our case), but when the WT bias is very high, the savings due to choosing the better method might be significant. Another option to reduce WT bias is to modify the base composition ratio during primer synthesis^57^, but this strategy may require multiple PCR optimization steps and may not be thus suitable for high-throughput experiments.

Another factor playing a role in library quality is the primer annealing bias. We modeled this bias only through an increased annealing probability of primers carrying the WT codon, relative to the other primers. However, the annealing bias may manifest itself more finely, according to the number of Watson-Crick pairing mismatches in a codon: the WT codon is an extreme case with zero mismatches, and it is possible that a codon with, say, one mismatch will be more likely to anneal than a codon with two mismatches, etc. Our statistical analysis did not detect such a phenomenon, but also did not rule it out. A much larger data set is required to study the existence and magnitude of this phenomenon.

In summary, we have presented a faster, more economical and reliable method for performing the QQC, based on liquid cultures. Importantly, the QQC should be combined with the Q-values to assess overall library quality. We also demonstrated that primer purity has a significant effect on library yield, but that some suppliers might offer primers of higher quality than others without additional purification steps. In addition, we provided guidelines for choosing optimally a randomization scheme, depending on the screening costs and other experimental parameters. Our guidelines also apply to any PCR-based method for library preparation, including combinatorial gene synthesis^58^, gene assembly^59^ and overlap extension PCR^60^.

## Additional Information

**How to cite this article**: Acevedo-Rocha, C. G. *et al*. Economical analysis of saturation mutagenesis experiments. *Sci. Rep*. **5**, 10654; doi: 10.1038/srep10654 (2015).

## Figures and Tables

**Table 1:**
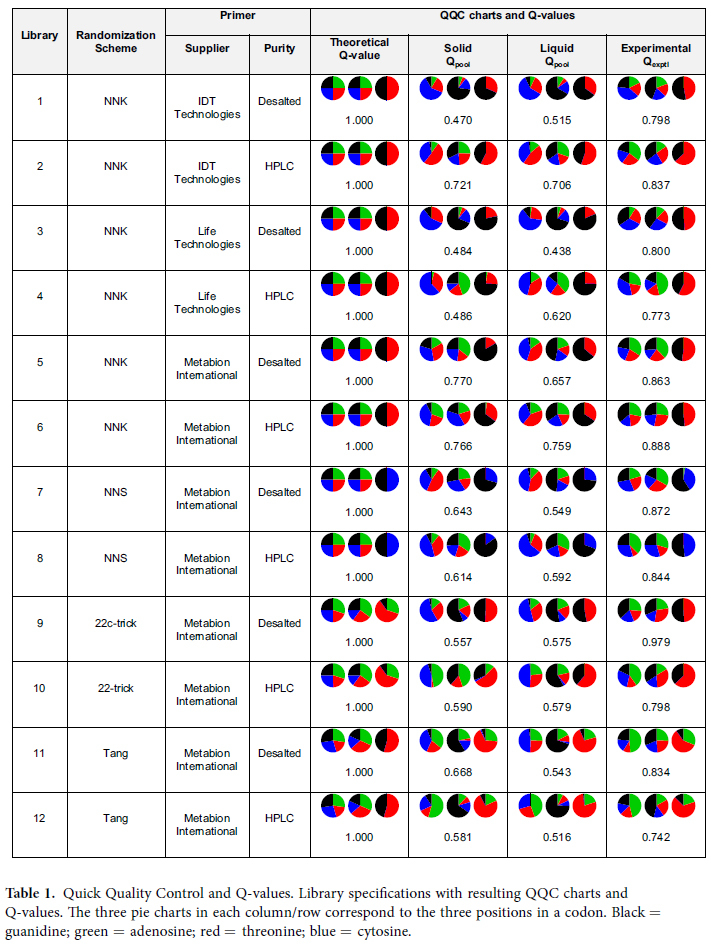
Quick Quality Control and Q-values. Library specifications with resulting QQC charts and Q-values. The three pie charts in each column/row correspond to the three positions in a codon. Black = guanidine; green = adenosine; red = threonine; blue = cytosine.

**Table 2:** Sequencing results summary obtained from 96 single colonies formed on agar plates per library.

Library	Successfully randomized	Yield (%)	>1 base per position	Non-target mutations	Primer misinsertions	Suboptimal sequencing	Missed amino acids
1	68	72.3	19	6	1	2	Met, Asp
2	65	68.4	24	6	-	1	Lys, Asn, His
3	55	59.8	29	5	3	4	Met, Lys, Asn, Phe
4	54	58.1	37	2	-	3	Ile
5	50	52.1	42	4	-	-	-
6	64	66.7	27	5	-	-	-
7	39	41.9	50	4	-	3	Met, Ile, Gln, Trp
8	64	67.4	24	6	1	1	Phe, Tyr
9	57	59.4	37	1	1	-	-
10	67	69.8	24	4	1	-	Asp, Tyr
11	51	53.7	41	1	2	1	Asp, Tyr
12	65	69.1	23	6	-	2	Lys, Asp, Tyr
